# Supplementary material for: Loss of Grem1-lineage chondrogenic progenitor cells causes osteoarthritis
Source: Nat Commun. 2023 Oct 31;14:6909. doi: 10.1038/s41467-023-42199-1 (PMC10618187; doi:10.1038/s41467-023-42199-1)
Supplement: Supplementary file 1 — Supplementary Information [file 41467_2023_42199_MOESM1_ESM.pdf]

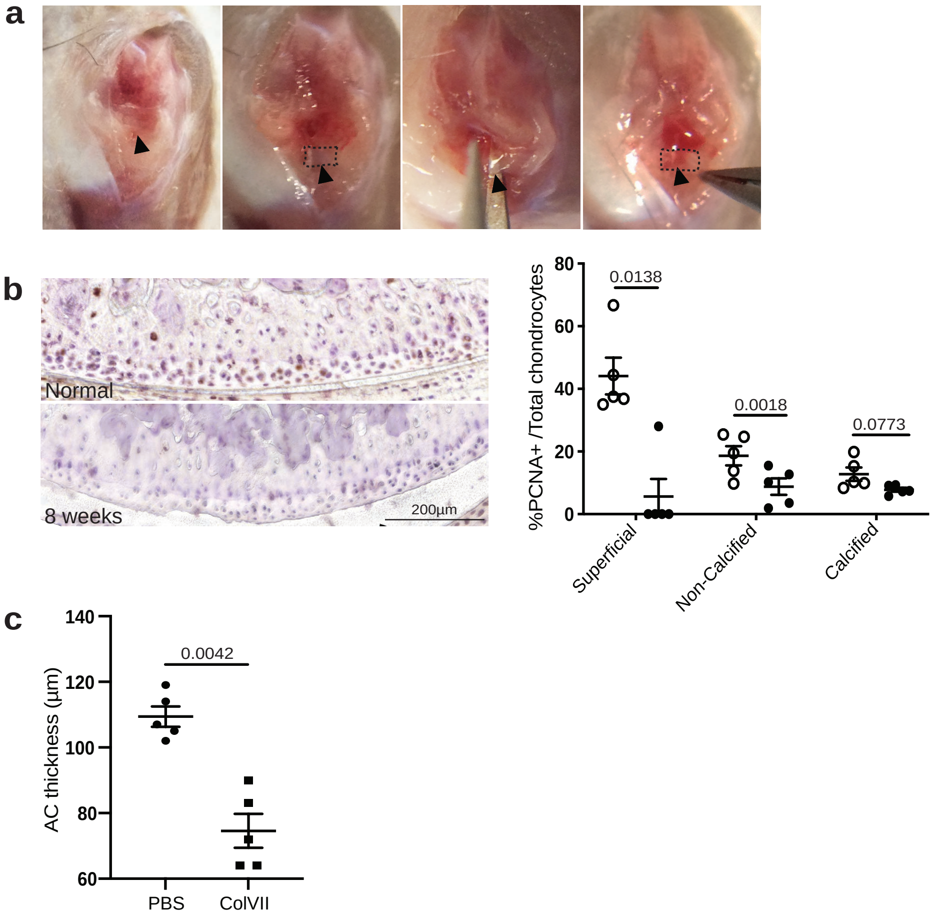

Supplementary Figure 1 | a, The destabilisation of the medial meniscus model (DMM) generates OA pathology through altered joint biomechanics, following surgical transection of the medial meniscus. Sequential images showing the transection of the medial meniscus within the knee joint (dotted box) to produce the DMM-induced OA pathology in mice. **b**, Representative images of PCNA immunohistochemistry in knee joints 8 weeks after DMM surgery (left) in comparison to paired, no surgery contralateral knee (normal). PCNA quantification shows a significant decrease in proliferating cells in both the superficial and non-calcified zones of the AC following DMM surgery (●) compared to no surgery (○), indicating no repair response 8 weeks post-surgery (right). n=5 animals per group, each data point represents an individual animal analysed. Paired, two-tailed t test. Bars denote s.e.m. **c**, Quantification of AC thickness in the ColVII induced OA model showing a significant decrease in cartilage thickness between the control (●) and ColVII treated (■) knees. n=5 animals per group, each data point represents an individual animal analysed. Unpaired, two-tailed t test. Source data are provided as a Source Data file.

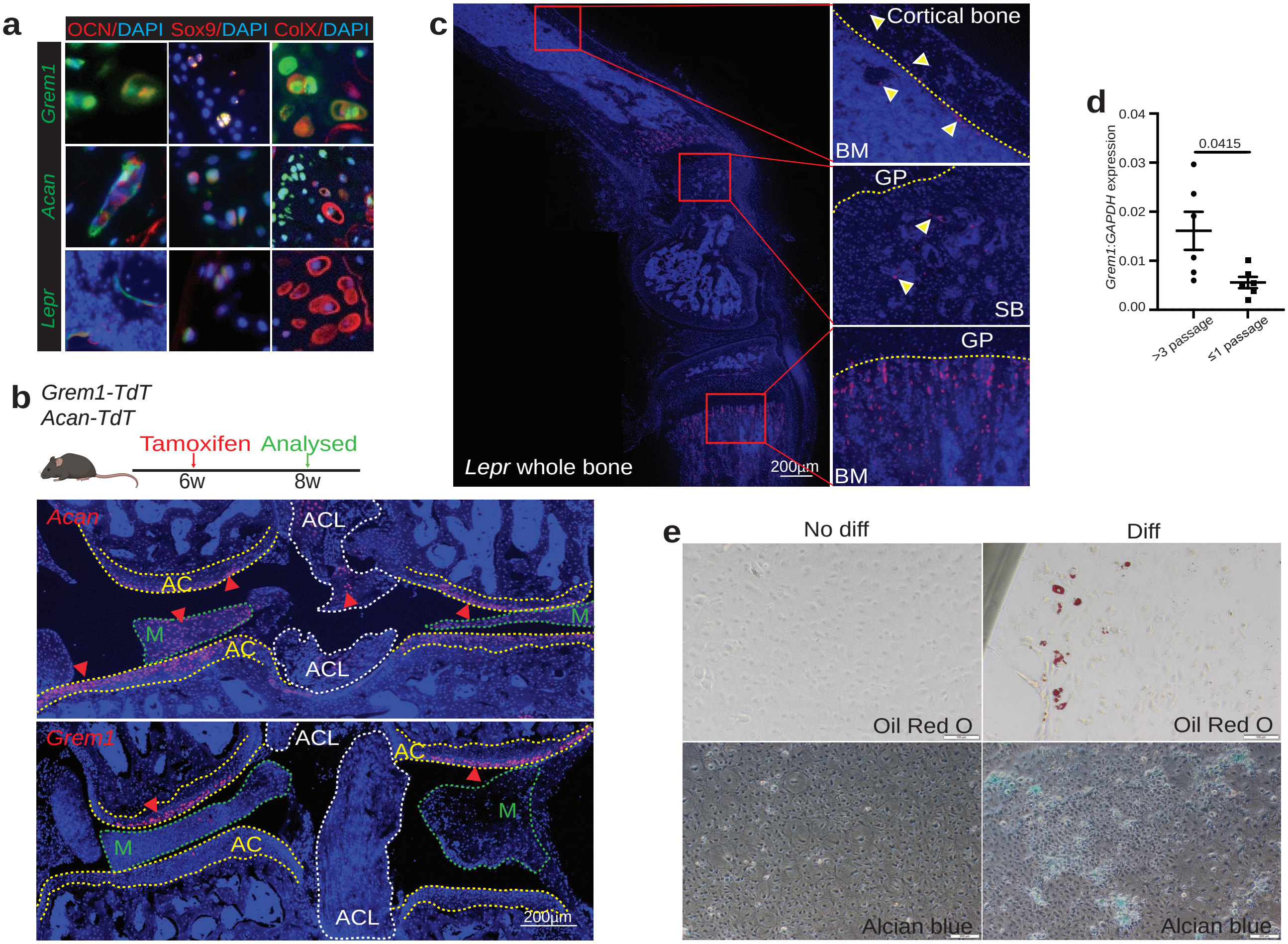

Supplementary Figure 2 | a, High magnification images of cells highlighted with yellow arrow in Figure 2c. **b**, Experimental Schema (top). Coronal sections of representative images of *Acan-TdT* (middle) and *Grem1-TdT* (bottom) joint depicting population of lineage traced cells. Meniscus, M, articular cartilage, AC, anterior cruciate ligament, ACL. **c**, Representative image of whole *LepR-TdT* bone at 8 weeks of age showing lineage tracing in cortical bone, subchondral bone (SB) and bone marrow (BM) just after the growth plate (GP) as indicated by arrows, but not the AC. **d**, Real time PCR showed a significantly higher expression of *Grem1* in *Acan* clones that were capable of expansion (●) compared to those that were not (■), suggesting *Grem1* expression correlates with stemness. Cells were pooled from n=3 mice to generate clones, n=6 clones analysed/group, each data point represents average data from 3 technical replicates for an individual clone isolated. Bars denote s.e.m., statistical analyses using two-sided t test with Welch's correction. **e,** Osteogenesis and chondrogenesis assays conducted using traditional MSC isolated from whole bone of C57Bl6 animals as positive experimental controls to show successful differentiation into adipogenic and chondrogenic cells using the same media. Source data are provided as a Source Data file. Mouse image in figure schema created with BioRender.com.

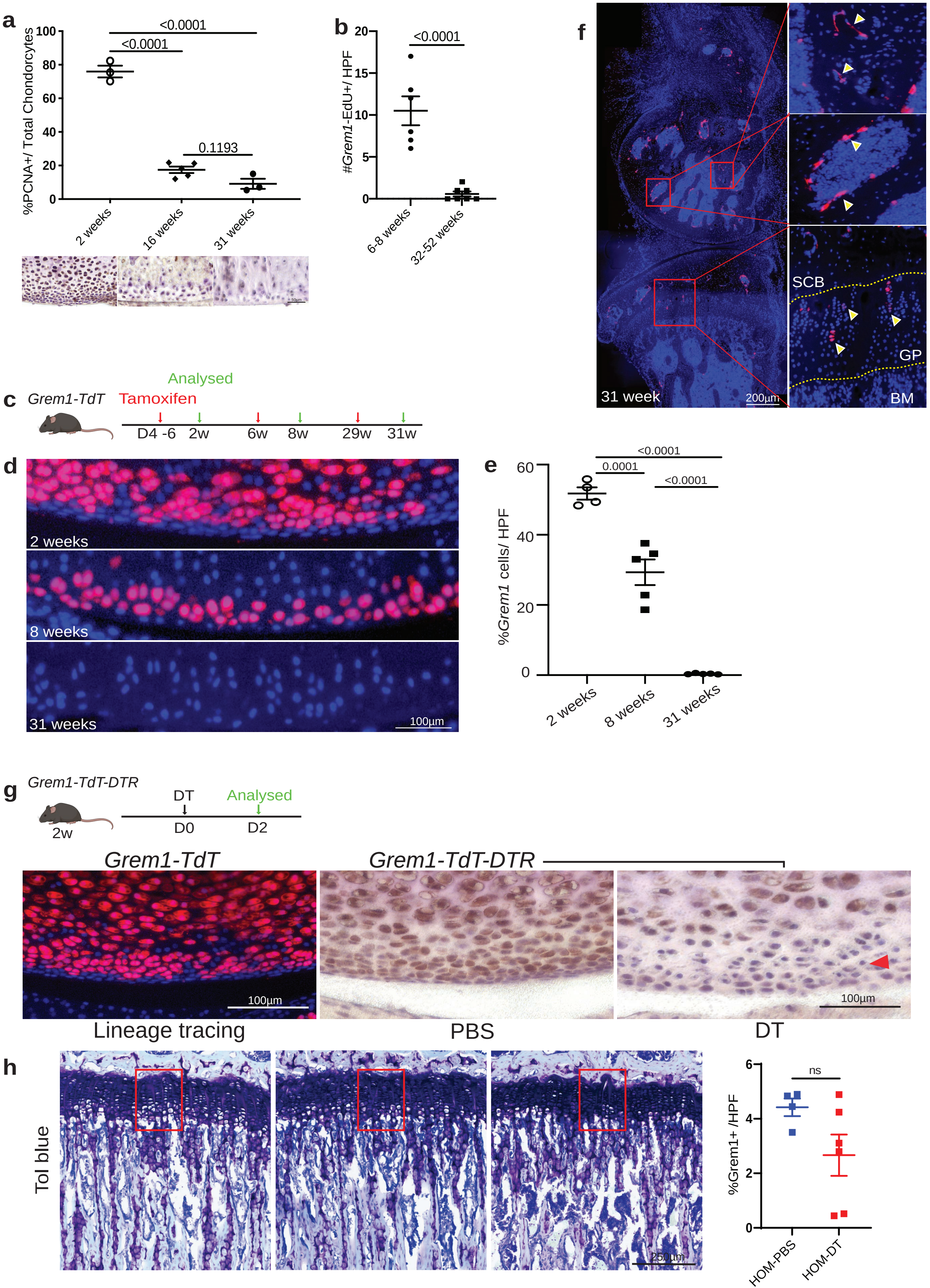

a
%PCNA+/ Total Chondrocytes
<0.0001
<0.0001
0.1193
2 weeks
16 weeks
31 weeks
b
#Grem1-EdU+/ HPF
<0.0001
6-8 weeks
32-52 weeks
f
SCB
GP
BM
31 week
200µm
c
Grem1-TdT
Analysed
Tamoxifen
D4 -6
2w
6w
8w
29w
31w
d
2 weeks
8 weeks
31 weeks
100µm
e
%Grem1 cells/ HPF
<0.0001
0.0001
<0.0001
2 weeks
8 weeks
31 weeks
g
Grem1-TdT-DTR
DT
Analysed
2w
D0
D2
Grem1-TdT
Grem1-TdT-DTR
100µm
100µm
Lineage tracing
PBS
DT
h
Tol blue
250µm
%Grem1+ /HPF
ns
HOM-PBS
HOM-DT

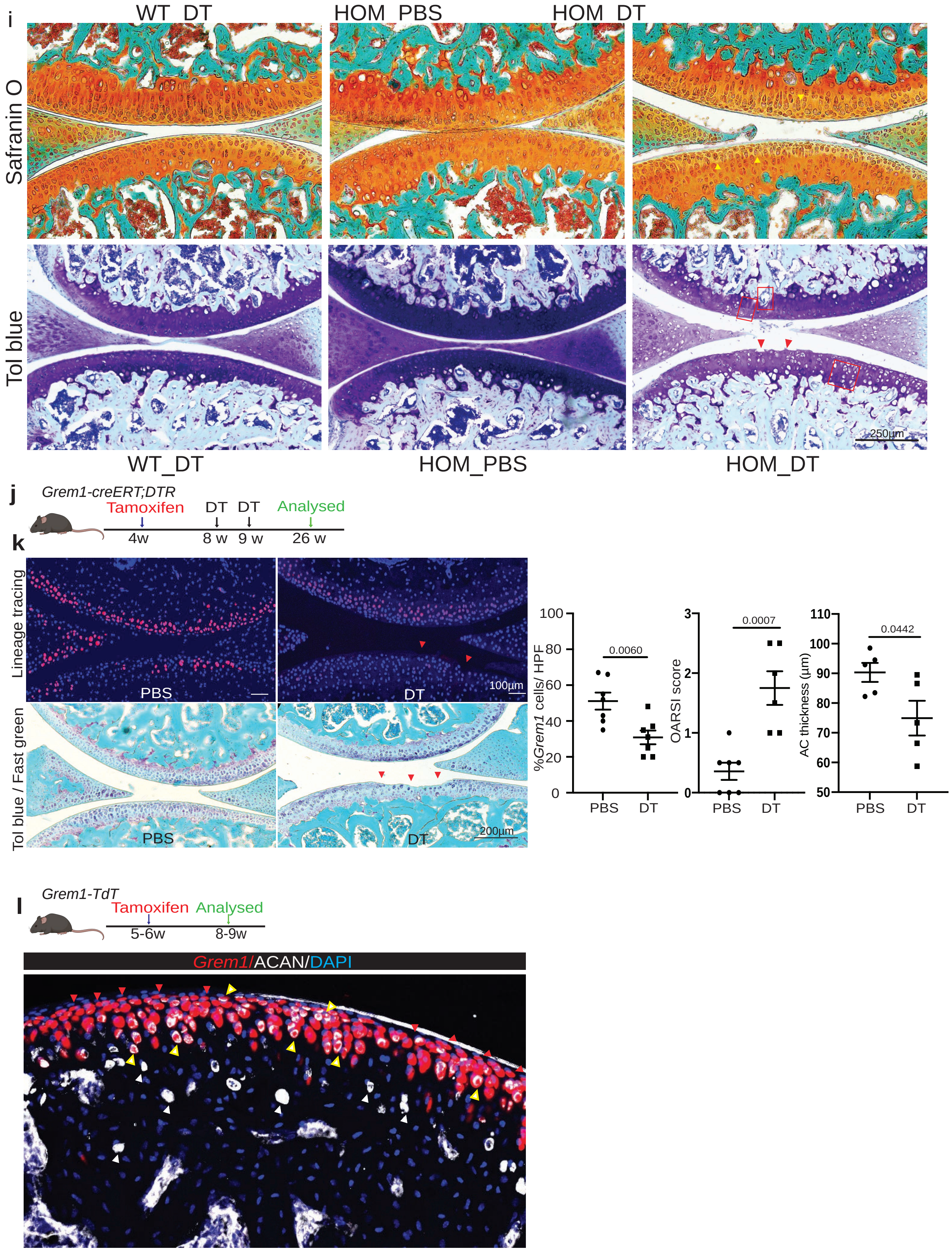
i
WT_DT
HOM_PBS
HOM_DT
Safranin O
Tol blue
250μm
WT_DT
HOM_PBS
HOM_DT
j
Grem1-creERT;DTR
Tamoxifen
DT
DT
Analysed
4w
8 w
9 w
26 w
k
Lineage tracing
PBS
DT
100μm
Tol blue / Fast green
PBS
DT
200μm
%Grem1 cells/ HPF
0.0060
PBS
DT
OARSI score
0.0007
PBS
DT
AC thickness (μm)
0.0442
PBS
DT
l
Grem1-TdT
Tamoxifen
Analysed
5-6w
8-9w
Grem1/ACAN/DAPI

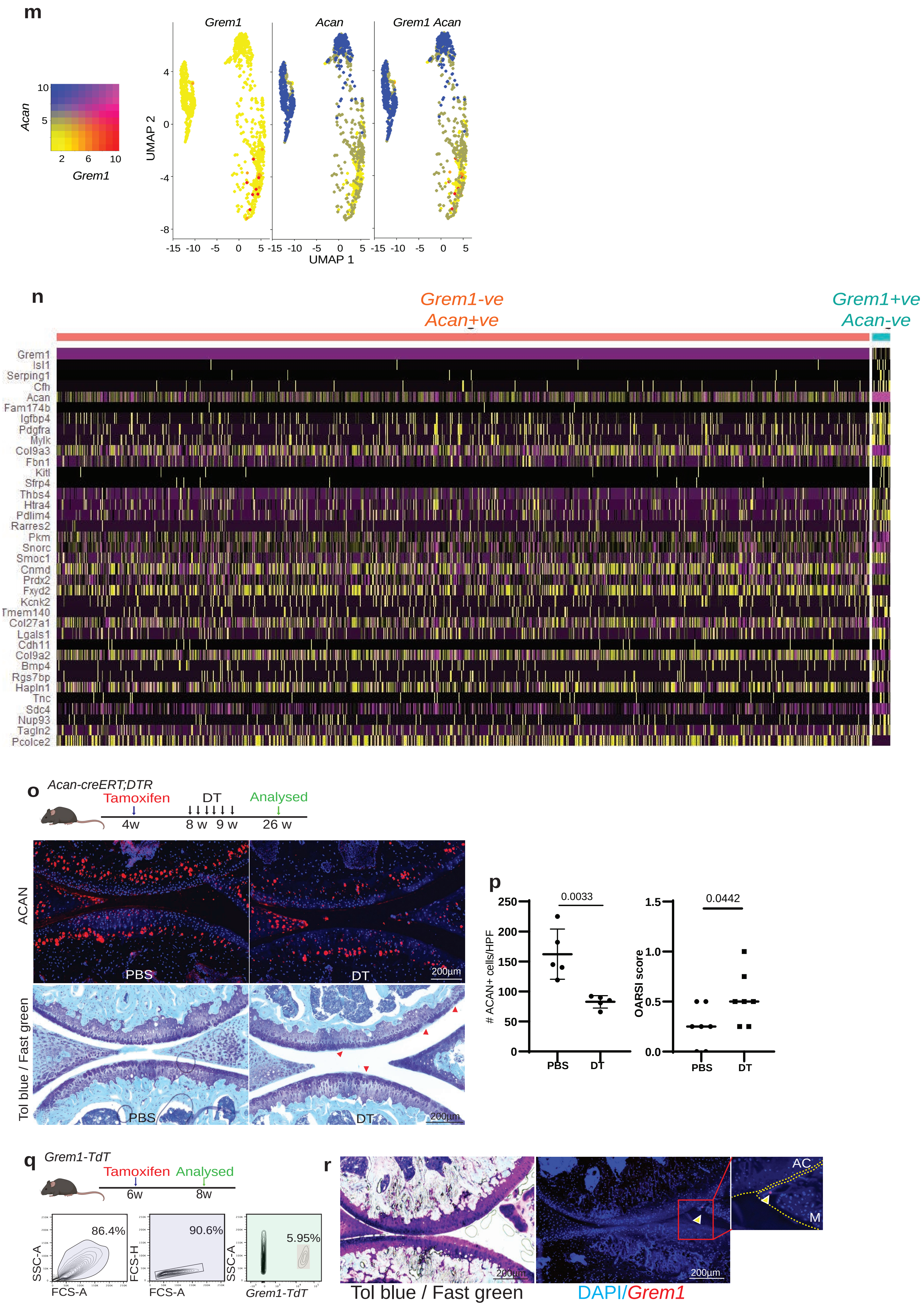
m
Grem1
Acan
Grem1 Acan
Acan
Grem1
UMAP 2
UMAP 1
n
Grem1-ve
Acan+ve
Grem1+ve
Acan-ve
Grem1
Isl1
Serping1
Cfh
Acan
Fam174b
Igfbp4
Pdgfra
Mylk
Col9a3
Fbn1
Kitl
Sfrp4
Thbs4
Htra4
Pdlim4
Rarres2
Pkm
Snorc
Smoc1
Cnmd
Prdx2
Fxyd2
Kcnk2
Tmem140
Col27a1
Lgals1
Cdh11
Col9a2
Bmp4
Rgs7bp
Hapln1
Tnc
Sdc4
Nup93
Tagln2
Pcolce2
o
Acan-creERT;DTR
Tamoxifen
DT
Analysed
4w
8 w 9 w
26 w
ACAN
PBS
DT
200µm
Tol blue / Fast green
p
ACAN+ cells/HPF
0.0033
PBS
DT
OARSI score
0.0442
q
Grem1-TdT
Tamoxifen
Analysed
6w
8w
86.4%
90.6%
5.95%
SSC-A
FCS-A
FCS-H
Grem1-TdT
r
AC
M
Tol blue / Fast green
DAPI/Grem1

Supplementary Figure 3 | a, Quantification of the percentage of PCNA positive cells in the AC at different postnatal stages showed a significant decrease in chondrocyte proliferation from postnatal developmental to adulthood. Representative images of PCNA staining (bottom). Each data point represents an individual animal analysed, n=3 mice for 2 weeks, n=5 for 16 weeks, and n=3 for 31 weeks, whole AC counted. One-way Anova Tukey's test. Bars denote s.e.m.
b, Quantification of the number of *Grem1*-EdU positive cells of mice in different stages of adulthood showing a significant decrease in proliferation with age. n=6 mice at 6-8 weeks, n=7 mice at 32-52 weeks. Unpaired, two-sided t test, bars denote st.dev.
c, Experiment schema. **d,** Representative images of *Grem1*-lineage articular cells within the articular joints of mice at different postnatal stages, quantified in e. **e,** Quantification of the total number of *Grem1*-lineage articular chondrocytes as a percentage of the total cells per high power field (HPF) in different postnatal stages showing a significant loss of *Grem1*-lineage AC cells in aged articular knee joints. n=4 mice at 2 weeks, n=5 mice at 8 weeks, n=5 at 31 weeks, each dot represents individual animal, 4 ROI per slide per sample. One-way Anova Tukey's test, bars denote s.e.m.
f, Representative image of a 31-week-old *Grem1-TdT* bone showing *Grem1* cells giving rise to osteoblasts within the subchondral bone space (SCB), few chondrocytes within the GP and almost none observed in the AC.
g, Experimental schema (top) Representative images of knee joint from 2-week-old lineage traced *Grem1-TdT* mouse (bottom left), in comparison to our new transgenic mouse line *Grem1-Td-DTR* treated with PBS and stained with anti-RFP to visualise *Grem1* cells (bottom middle) or *Grem1-Td-DTR* mice treated with 250ng of DT also stained with anti-RFP (bottom right). In both *Grem1-TdT* and *Grem1-Td-DTR* mice the *Grem1* cells are distributed identically in the developing joint and targeted ablation of these cells with DT treatment in *Grem1-Td-DTR* mice is indicated with red arrow.
h, Representative images of the growth plate in early adult mice treated with PBS or DT (mice treated as in Figure 3c) stained with tol blue, showed no significant growth plate changes in sex and age matched samples. Images from *Grem1-Td-DTR* wildtype (WT) and homozygous (HOM) mice treated with PBS or DT. Quantification of the total *Grem1*-lineage cells in the GP as a percentage of total GP chondrocytes showed no significant depletion with this DT regimen. Welch's two-tailed t test. n=4 mice for PBS, n=6 mice for DT, data point represents an individual animal analysed.

i, Representative images of *Grem1-Td-DTR* joints from WT treated with DT, and HOM treated with PBS or DT, stained with Safranin O (top) or Tol Blue (bottom) and fast green showing proteoglycan loss as indicated by decreased staining intensity with the orange (Safranin O, yellow arrowheads) and purple (Tol Blue) stains in HOM DT-treated animals. HOM DT-treated also showed signs of AC damage indicated by red arrows. Red boxes represent OA pathology indicated in Fig 3g-k.
j, Experimental schema. **k**, Representative images of distal femur joint from *Grem1-creRET;DTR* mice treated with PBS or DT (top) showing loss of *Grem1* AC cells with DT and stained with Toluidine blue and Fast green with red arrows indicating lesions indicative of OA pathology (bottom). n=7 mice per group. Quantification of the total number of *Grem1* AC cells per HPF showing a significant loss of *Grem1* cells in the AC in DT treated mice (left). Unblinded histopathological assessment using OARSI grading showed a significantly increased OA severity when *Grem1* cells are ablated in the joint (DT, square, n=6 mice) compared to PBS (circle, n=7) control (middle). Quantification of AC thickness in *Grem1-creERT;DTR* mice treated with DT (square, n=5 mice) or contralateral joint with PBS (circle, n=5 mice) as control (right). Data point represents an individual animal analysed. Unpaired, two-tailed t test, bars denote s.e.m.
l, Experimental schema and representative image of ACAN expression (white) in *Grem1*-lineage (red) cells in the AC, but also showing ACAN+ only and *Grem1*-lineage only marked cells. White arrows indicate ACAN+ only cells, red arrows *Grem1*-lineage only, yellow arrows both ACAN+ and *Grem1*-lineage cells. n=2 mice.
m, Single cell RNA (scRNA) sequencing of *Grem1*-lineage articular cells from mouse knee highlighting only *Grem1*-expressing (*Acan* negative) and *Acan*-expressing (*Grem1* negative) subpopulations from the dataset. Data from these cells was used to analyse differentially expressed genes between these two populations as shown in Supplementary Figure 3n.
n, heat map depicting significantly differentially expressed transcripts between *Grem1*-lineage articular cells expressing *Grem1* but not *Acan* (*Grem1+ve Acan –ve*, blue), and those expressing *Acan* but not *Grem1* (*Grem1-ve Acan +ve*, red) in the early adult knee (p adj. $p<0.05$).
o, Experimental schema (top). Representative images of AC knee joint from *Acan-creERT;DTR* mice treated with PBS or DT (top) showing loss of ACAN-expressing AC cells with DT, identified using ACAN immunostaining, and stained with Toluidine blue and Fast green with red arrows indicating lesions indicative of OA pathology (bottom). n=5 mice per group.
p, Quantification of the total number of ACAN-expressing AC cells per HPF showing a significant loss of ACAN+ cells in the AC in DT treated mice (left, n=5 mice/group). Unblinded histopathological assessment using OARSI grading showed a mild but significant increase in OA severity when ACAN-expressing cells are ablated in the joint compared to PBS control (right, n=7 mice/group. Data point represents an individual animal analysed. Unpaired, two-sided t test. Bars denote st.dev.
q, Experimental schema (top). FACS plot showing *Grem1*-lineage cells sorting strategy (bottom).
r, Representative images of DMM knee joints implanted with *Grem1*-lineage sorted cells via intra-articular injection. Serial sections of joint stained with Tol blue and fast green (left) and immunofluorescence image (right) showing *Grem1*-lineage cells homing to the meniscus (yellow arrow).
Source data are provided as a Source Data file. Mouse image in figure schemas created with BioRender.com.

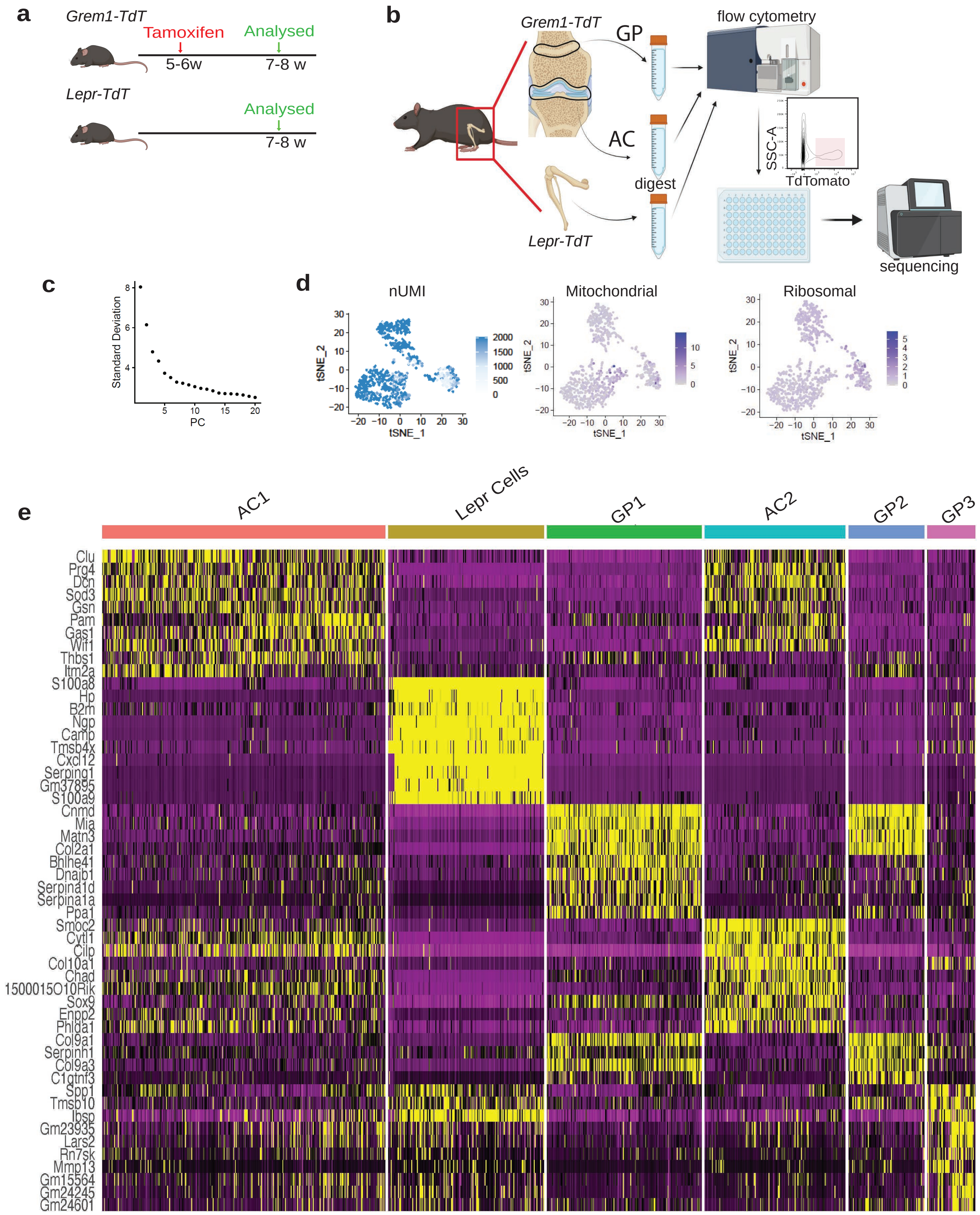
a
Grem1-TdT
Tamoxifen
Analysed
5-6w
7-8 w
Lepr-TdT
Analysed
7-8 w
b
Grem1-TdT
GP
AC
digest
Lepr-TdT
flow cytometry
SSC-A
TdTomato
sequencing
c
Standard Deviation
PC
d
nUMI
Mitochondrial
Ribosomal
tSNE_1
tSNE_2
e
AC1
Lepr Cells
GP1
AC2
GP2
GP3
Clu
Prg4
Dcn
Sod3
Gsn
Pam
Gas1
Wif1
Thbs1
Itm2a
S100a8
Hp
B2m
Ngp
Camp
Tmsb4x
Cxcl12
Serping1
Gm37895
S100a9
Cnmd
Mia
Matn3
Col2a1
Bhlhe41
Dnajb1
Serpina1d
Serpina1a
Ppa1
Smoc2
Cytl1
Cilp
Col10a1
Chad
1500015O10Rik
Sox9
Enpp2
Phlda1
Col9a1
Serpinh1
Col9a3
C1qtnf3
Spp1
Tmsb10
Ibsp
Gm23935
Lars2
Rn7sk
Mmp13
Gm15564
Gm24245
Gm24601

f

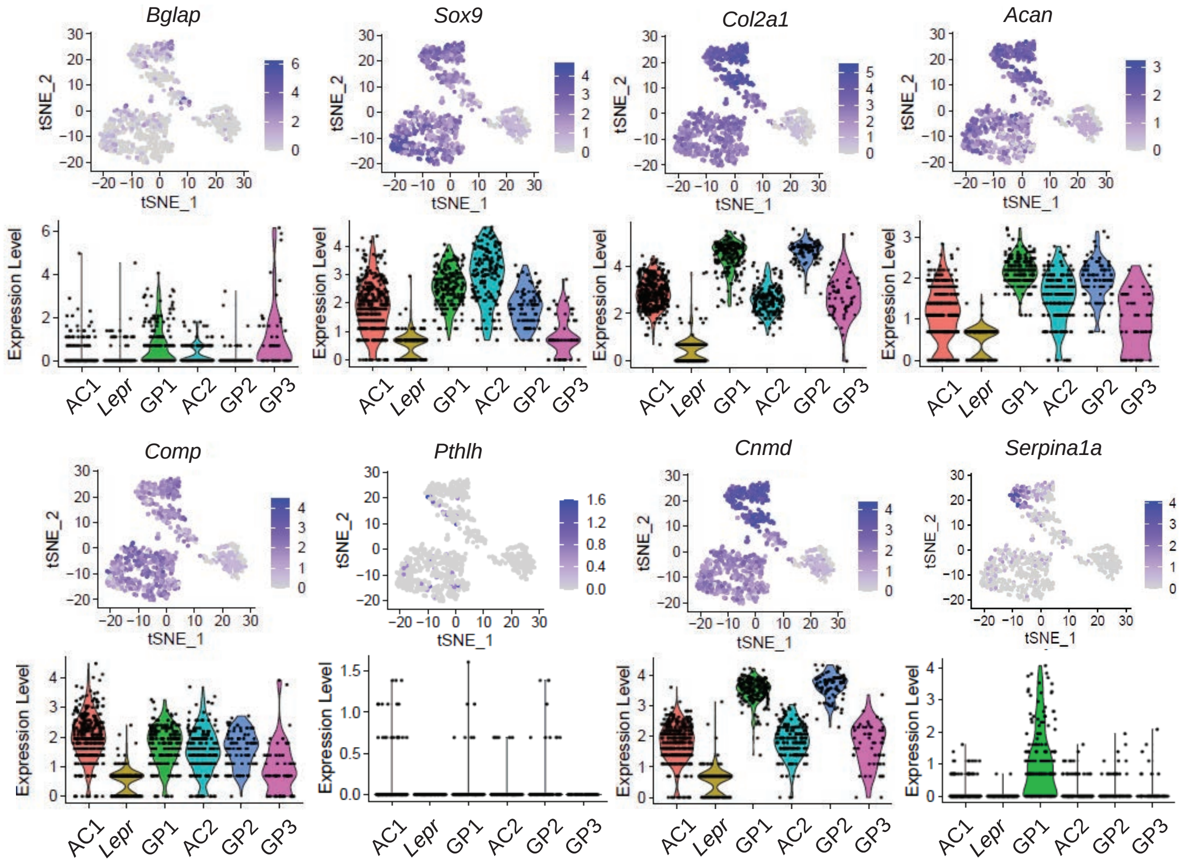
Bglap
Sox9
Col2a1
Acan
Comp
Pthlh
Cnmd
Serpina1a
tSNE_1
tSNE_2
Expression Level
AC1
Lepr
GP1
AC2
GP2
GP3

g

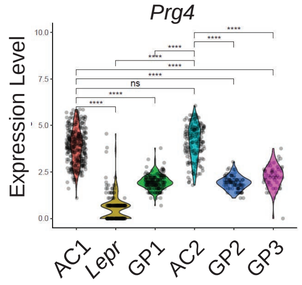
Prg4
Expression Level
ns
AC1
Lepr
GP1
AC2
GP2
GP3

h

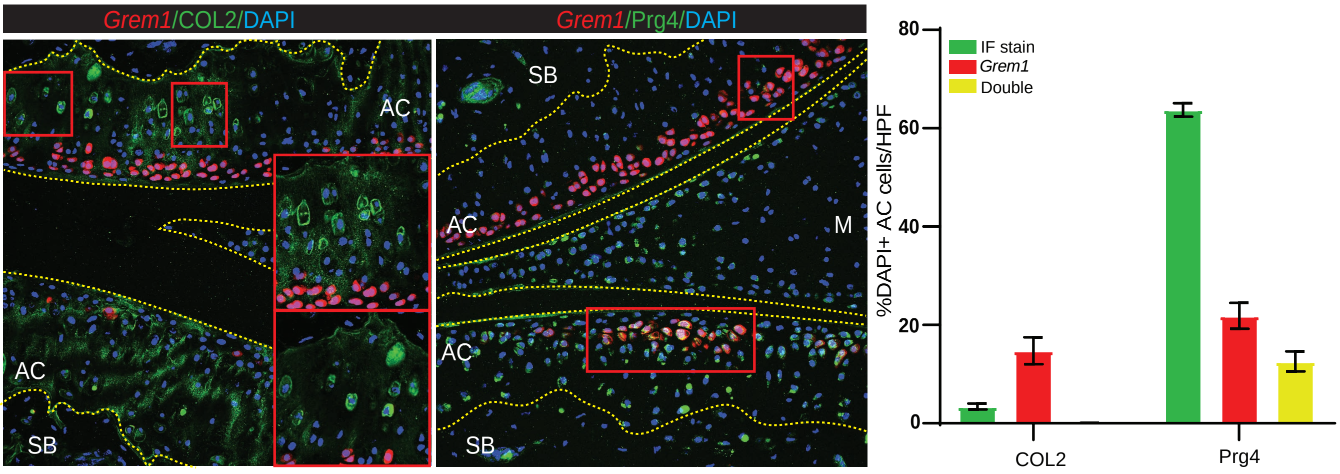
Grem1/COL2/DAPI
Grem1/Prg4/DAPI
AC
SB
M
IF stain
Grem1
Double
%DAPI+ AC cells/HPF
COL2
Prg4

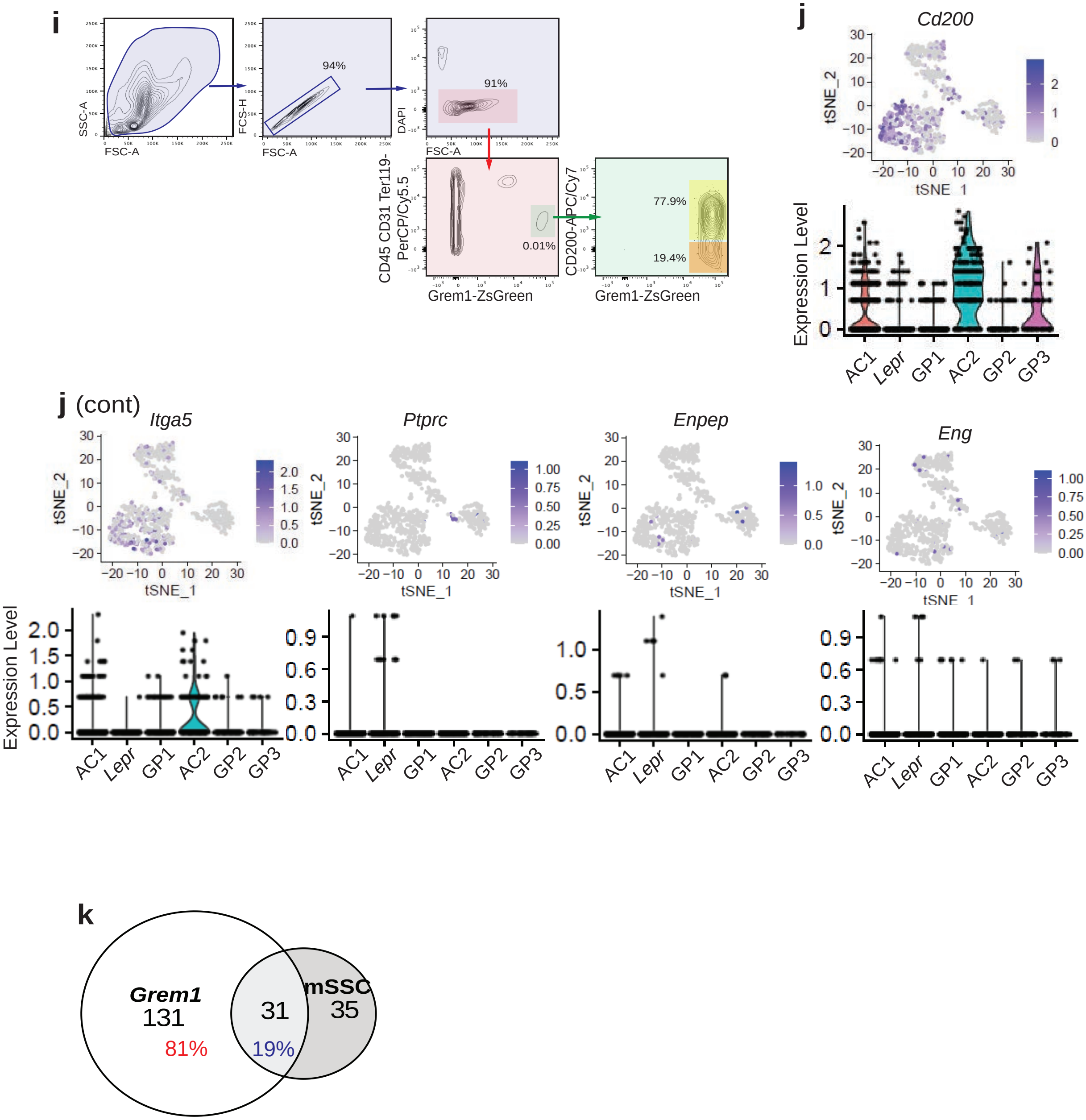
i
SSC-A
FSC-A
94%
FCS-H
DAPI
91%
FSC-A
CD45 CD31 Ter119-
PerCP/Cy5.5
0.01%
Grem1-ZsGreen
CD200-APC/Cy7
77.9%
19.4%
Grem1-ZsGreen
j
Cd200
tSNE_2
tSNE_1
Expression Level
AC1
Lepr
GP1
AC2
GP2
GP3
j (cont)
Itga5
Ptprc
Enpep
Eng
k
Grem1
131
81%
31
19%
mSSC
35

Supplementary Figure 4 | a, Experimental schema showing tamoxifen dosing and analysis schedule and **b**, subsequent dissection of AC, GP or whole bone tissue from *Grem1-TdT* and *LepR-TdT* animals, separately dissociated by digestion, then for each tissue source separately subjected to FACS isolation of TdT positive stem/precursor cell populations, before scRNA sequencing. Created with BioRender.com.

c, Elbow plot used to determine appropriate number of principal components (PC) used in scRNAseq data clustering to capture the majority of variation in the data.

d, scRNAseq quality control plots showing read depth is appropriate with the majority of unique molecular identifiers (UMI) >1000 (left), low mitochondrial (centre) and ribosomal RNA (right) content.

e, Heat map depicting unsupervised clustering of top 10 differentially expressed transcripts between the different scRNAseq clusters as shown in Figure 4b but here displayed with gene names.

f, Expression of classical osteoblast (osteocalcin, *Bglap)*, chondrocyte (*Sox9, Col2a1, Acan, Comp, Cnmd*) and growth plate markers (*Pthlh*) in the scRNAseq dataset.

g, Expression of AC marker, *Prg4* (encoding Lubricin), is significantly increased in the two AC clusters in comparison to the GP and *Lepr* cell populations, **** $p<0.0001$ Tukey's test.

h, Representative images of IF staining of *Grem1-TdT* joints induced at 6 weeks and collected at 8 weeks old (n=5 mice). Red squares highlight regions of interest with positive staining. COL2 staining of the AC showed little overlap between COL2 expressing chondrocytes and *Grem1*-lineage cells (yellow). PRG4 staining of the AC however, showed a larger population of *Grem1*-lineage cells expressing PRG4 (yellow). Quantification of total COL2, PRG4 and Grem1-lineage cells represented as a percentage of total AC chondrocytes, 4 high-powered fields analysed per slide. Only cells that were positive for DAPI were counted to ensure that only live cells were quantified. The percentage of *Grem1*-lineage cells that express COL2 was <0.1%, indicative of 2 distinct populations of cells. 64% of AC chondrocytes expressed PRG4 and about half of the *Grem1*-lineage cells also express PRG4. Quantification performed using n=5 animals per group.

i, Immunophenotyping of *Grem1*-lineage cells from total bone preps of 8-week-old mice using flow cytometry showed that 77.9% express the key mSSC marker CD200. n=3 animals.

j, tSNE and violin plots depict expression of transcripts encoding additional immunophenotypic markers of AlphaV+CD200+CD45-6C3-CD105-Ter-119-Tie2-Thy- mSSC cells in the scRNAseq data from *Grem1*- and *Lepr*-lineage cells. *Itga5* encodes AlphaV, *Ptprc* encodes CD45, *Enpep* encodes 6C3, *Eng* encodes CD105, while transcripts for *Ly76* (encoding Ter119), *Tek* (encoding Tie2) and *Thy1* (encoding Thy) were not detected.

k, Of the *Grem1*-lineage AC population that express *Grem1* in the scRNAseq data, only 19% also expressed mSSC immunophenotype genes, indicating most *Grem1*-expressing AC cells are likely distinct from mSSCs.

Source data are provided as a Source Data file. Mouse image in figure schemas created with BioRender.com.

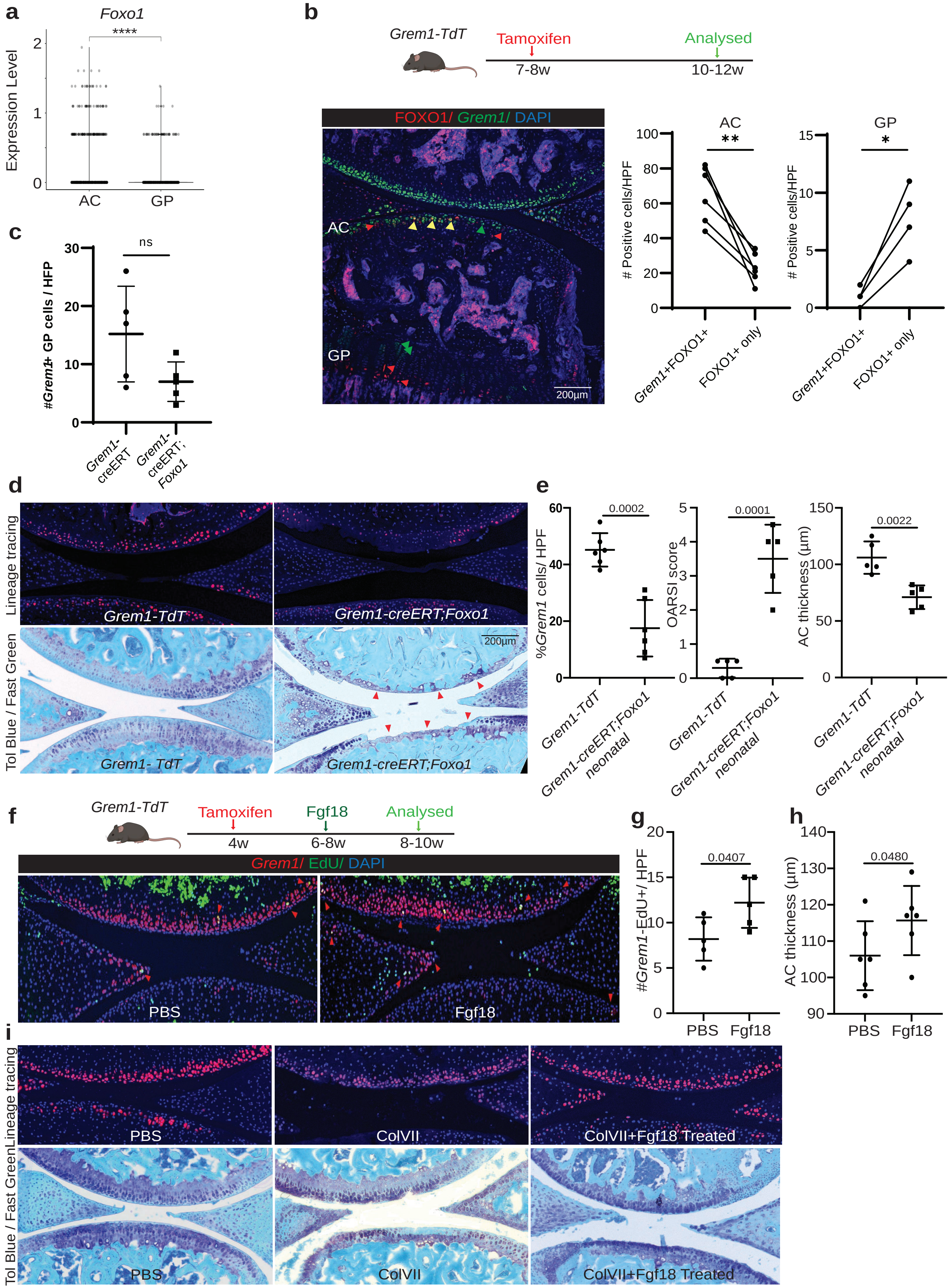
a
Foxo1
Expression Level
****
AC
GP
b
Grem1-TdT
Tamoxifen
Analysed
7-8w
10-12w
FOXO1/ Grem1/ DAPI
AC
GP
200µm
Positive cells/HPF
AC
**
GP
*
Grem1+FOXO1+
FOXO1+ only
c
ns
#Grem1+ GP cells / HFP
Grem1-creERT
Grem1-creERT;Foxo1
d
Lineage tracing
Tol Blue / Fast Green
Grem1-TdT
Grem1-creERT;Foxo1
200µm
e
0.0002
0.0001
0.0022
%Grem1 cells/ HPF
OARSI score
AC thickness (µm)
Grem1-TdT
Grem1-creERT;Foxo1 neonatal
f
Grem1-TdT
Tamoxifen
Fgf18
Analysed
4w
6-8w
8-10w
Grem1/ EdU/ DAPI
PBS
Fgf18
g
#Grem1-EdU+/ HPF
0.0407
h
AC thickness (µm)
0.0480
PBS
Fgf18
i
Lineage tracing
Tol Blue / Fast Green
PBS
ColVII
ColVII+Fgf18 Treated

Supplementary Figure 5

a, Expression of *Foxo1* in pooled articular cartilage (AC) or growth plate (GP) *Grem1*-lineage populations in scRNAseq dataset (described in **Figure 4, Supplementary Figure 4**). Tukey's test, **** $p<0.0001$.

b, Experimental schema and representative image of FOXO1 expression (red) in *Grem1*-lineage (green) cells in the AC but not GP. Red arrows indicate FOXO1+ only cells, green arrows *Grem1*-lineage only, yellow arrows both FOXO1+ and *Grem1*-lineage cells. Quantification of fluorescently labelled average cell number in the AC and GP to the right, n=6 mice per group, 2-4 high-powered fields of view (HPF)/ animal; two-sided t-test, *$p<0.05$, **$p<0.01$.

c, Quantification of the number of *Grem1*-lineage cells in the GP of *Grem1-TdT-Foxo1* deletion mice compared to *Grem1-TdT* mice showed no significant (ns) difference, schema for experiment depicted in **Fig 4.** n=5 mice per group, 2-3 HPF/animal; two-sided t-test. Bars denote st.dev.

d, Representative images of neonatally induced deletion of *Foxo1* in *Grem1-TdT* cells showing significant loss of AC (arrows).

e, Quantification of the percentage of *Grem1*-lineage articular chondrogenic progenitor (CP) cells per HPF showed a significant decrease in *Grem1*-lineage CP cells in neonatal induced *Grem1-TdT-Foxo1* mice (■) compared to *Grem1-TdT* mice (●) (left, n=6 mice/group). Unblinded histopathological scoring of OA pathology also showed a significant increase in OARSI score in *Grem1-TdT-Foxo1* deletion mice (■) compared to *Grem1-TdT* mice (●) (middle, n=5 mice/group), and AC thickness indicating the important role of *Grem1*-lineage CP cells in early AC development through *Foxo1* signalling (right, n=5 mice/ group). Each data point represents an individual animal analysed, bars denote st.dev. Unpaired, two-sided t-test.

f, Experimental schema (top). Representative IF image of EdU staining showing an increase in proliferating *Grem1*-lineage articular CP cells indicated by red arrows with FGF18 treatment (bottom). **g,** Quantification of the number of EdU positive *Grem1*-lineage articular CP cells showed that FGF18 treatment (■) significantly increased *Grem1*-lineage articular CP cell proliferation compared to PBS control ☎). n=5 mice/group, bars denote st.dev. Unpaired, two-sided t-test.

h, This increase in proliferation likely resulted in the increase in AC thickness in *Grem1*-TdT mice treated with FGF18 (■) compared to contralateral knee treated with PBS as control (●). n=5 mice/group. Statistical analyses used unpaired two-sided t test.

i, Experimental schema as in Fig.4h. Additional representative images of joints from *Grem1*-lineage control (PBS) mice or mice with ColVII induced OA, with or without FGF18 treatment (fluorescence, top), toluidine blue and fast green stained showing proteoglycan loss (bottom).

Source data are provided as a Source Data file. Mouse image in figure schemas created with BioRender.com.

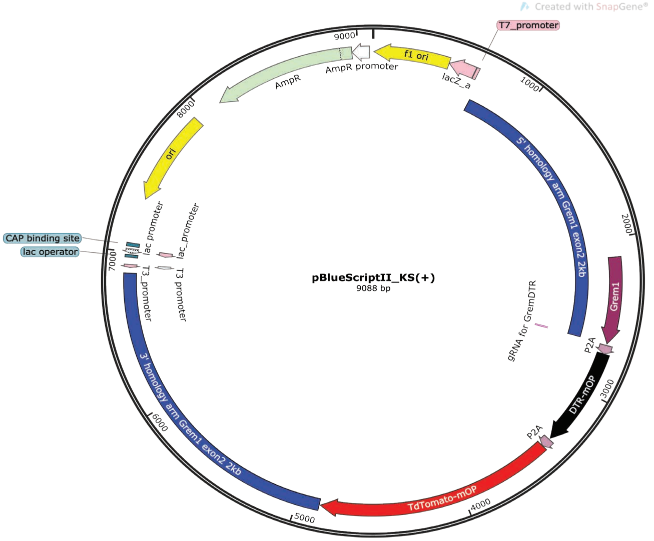

Supplementary Figure 6 | Diagram of plasmid construct for generation of *Grem1-TdT-DTR* knock-in mouse strain.

Supplementary Table 1.

Cluster	Top 10 Cluster defining transcripts
AC1	*Clu,Prg4,Sod3, Gsn, Pam, Itm2a, Cytl1,Cilp,Chad, Phlda1.*
LepR	*S100a8, Hp,B2m, Ngp, Camp,Tmsb4x, Cxcl12,Serping1,Gm37895,S100a9.*
GP1	*Cnmd,Mia,Matn3,Col2a1,Bhlhe41,Dnajb1,Serpina1d, Serpina1a,Ppa, Col9a1.*
AC2	*Smoc2,Cytl1,Cilp,Col10a1,Chad,1500015O10Rik,Sox9,Enpp2,Phlda1,Prg4.*
GP2	*Col9a1,Serpinh1,Col9a3,Cnmd,C1qtnf3,Col2a1,Matn3,Mia,Eno1b,Eno1.*
GP3	*Spp1,Gm23935,Lars2,Rn7sk,Gm23037,Bglap3,Bglap2,Bglap,Col1a1,Col1a2.*
