# Supplementary material for: Loss of Grem1-lineage chondrogenic progenitor cells causes osteoarthritis
Source: Nat Commun. 2023 Oct 31;14:6909. doi: 10.1038/s41467-023-42199-1 (PMC10618187; doi:10.1038/s41467-023-42199-1)
Supplement: Supplementary file 3 — Reporting Summary [file 41467_2023_42199_MOESM3_ESM.pdf]

nature portfolio

Corresponding author(s): A/Prof Siddhartha Mukherjee, A/Prof Susan Woods, A/Prof Daniel Worthley

Last updated by author(s): 2023/09/07

Reporting Summary

Nature Portfolio wishes to improve the reproducibility of the work that we publish. This form provides structure for consistency and transparency in reporting. For further information on Nature Portfolio policies, see our Editorial Policies and the Editorial Policy Checklist.

Statistics

For all statistical analyses, confirm that the following items are present in the figure legend, table legend, main text, or Methods section.

n/a	Confirmed	
☐	☒	The exact sample size (*n*) for each experimental group/condition, given as a discrete number and unit of measurement
☐	☒	A statement on whether measurements were taken from distinct samples or whether the same sample was measured repeatedly
☐	☒	The statistical test(s) used AND whether they are one- or two-sided<br>*Only common tests should be described solely by name; describe more complex techniques in the Methods section.*
☒	☐	A description of all covariates tested
☒	☐	A description of any assumptions or corrections, such as tests of normality and adjustment for multiple comparisons
☐	☒	A full description of the statistical parameters including central tendency (e.g. means) or other basic estimates (e.g. regression coefficient) AND variation (e.g. standard deviation) or associated estimates of uncertainty (e.g. confidence intervals)
☐	☒	For null hypothesis testing, the test statistic (e.g. *F*, *t*, *r*) with confidence intervals, effect sizes, degrees of freedom and *P* value noted<br>*Give P values as exact values whenever suitable.*
☒	☐	For Bayesian analysis, information on the choice of priors and Markov chain Monte Carlo settings
☒	☐	For hierarchical and complex designs, identification of the appropriate level for tests and full reporting of outcomes
☒	☐	Estimates of effect sizes (e.g. Cohen's *d*, Pearson's *r*), indicating how they were calculated

Our web collection on statistics for biologists contains articles on many of the points above.

Software and code

Policy information about availability of computer code

Data collection	Flow cytometry was performed on FACSFusion Cell sorter or LSR Fortessa (BD Biosciences). Flo Jo (vl0.6.2) was used for data acquisition,
Data analysis	Image J software (v2.1.0/1.53d) was used to stitch together multiple images to form picture of the whole bone. Statistical analyses were performed using Prism 8/9 (GraphPad software Inc.) and scRNAseq analysis using the following functions in the base or Seurate packages (version 3.2.2 or v5) in the R environment (version 4.0.2 or 4.3.1):FindClusters(),FindAllMarkers(),VlnPlot(), chisq.test(). No new software was developed for this manuscript. Image analysis using ImageJ v2. .O/l.53d (NIH,http://imagej.nih.gov/ij/), FACS analysis was conducted using FlowJo vl0.6.2 software.

For manuscripts utilizing custom algorithms or software that are central to the research but not yet described in published literature, software must be made available to editors and reviewers. We strongly encourage code deposition in a community repository (e.g. GitHub). See the Nature Portfolio guidelines for submitting code & software for further information.

Data

Policy information about availability of data

All manuscripts must include a data availability statement. This statement should provide the following information, where applicable:

- Accession codes, unique identifiers, or web links for publicly available datasets
- A description of any restrictions on data availability
- For clinical datasets or third party data, please ensure that the statement adheres to our policy

The scRNAseq data sets from this manuscript have been deposited in GEO, accession numbers (GSE193175 at https://www.ncbi.nlm.nih.gov/geo/query/acc.cgi?acc=GSE193175 and GSE242732 at https://www.ncbi.nlm.nih.gov/geo/query/acc.cgi?acc=GSE242732).

Research involving human participants, their data, or biological material

Policy information about studies with human participants or human data. See also policy information about sex, gender (identity/presentation), and sexual orientation and race, ethnicity and racism.

Reporting on sex and gender	This study did not include human participants
Reporting on race, ethnicity, or other socially relevant groupings	This study did not include human participants
Population characteristics	This study did not include human participants
Recruitment	This study did not include human participants
Ethics oversight	This study did not include human participants

Note that full information on the approval of the study protocol must also be provided in the manuscript.

Field-specific reporting

Please select the one below that is the best fit for your research. If you are not sure, read the appropriate sections before making your selection.

☒ Life sciences ☐ Behavioural & social sciences ☐ Ecological, evolutionary & environmental sciences

For a reference copy of the document with all sections, see nature.com/documents/nr-reporting-summary-flat.pdf

Life sciences study design

All studies must disclose on these points even when the disclosure is negative.

Sample size	No statistical test was used to pre-determine the sample sizes. Sample sizes were based on experience from previous work with assays performed (Worthley et al., 2015, Cell 160:269-284). For assays with commonly high variability we typically used n≥5 and for assays with commonly low variability we typically used n=3-5.
Data exclusions	No data was excluded.
Replication	All data presented are biological replicates unless otherwise stated in the figure legends. Each experimental finding was reproduced in at least two independent experiments.
Randomization	Animals were allocated randomly into the different experimental group.
Blinding	Investigators were not blinded during data collection and analysis, except for blinded scoring of OA histopathology in Grem1-Td-DTR mice . Controls and samples were treated equally.

Reporting for specific materials, systems and methods

We require information from authors about some types of materials, experimental systems and methods used in many studies. Here, indicate whether each material, system or method listed is relevant to your study. If you are not sure if a list item applies to your research, read the appropriate section before selecting a response.

Materials & experimental systems

n/a	Involved in the study
☐	☒ Antibodies
☒	☐ Eukaryotic cell lines
☒	☐ Palaeontology and archaeology
☐	☒ Animals and other organisms
☒	☐ Clinical data
☒	☐ Dual use research of concern
☒	☐ Plants

Methods

n/a	Involved in the study
☒	☐ ChIP-seq
☐	☒ Flow cytometry
☒	☐ MRI-based neuroimaging

Antibodies

Antibodies used

Immunohistochemistry & immunofluorescent antibodies: anti-PCNA (#ab18197 Abcam, 1:200), ColX (#ab58632 Abcam 1:200), OCN (#ab93876 Abcam, 1:200), Lubricin/PRG4 (#ab28484 Abcam, 1:250), COL2 (#ab34712 Abcam, 1:250), FOXO1 (#MA5-32114 ThermoFisher Invitrogen, 1:150), Sox9 (#AB5535 Millipore, 1:400), ACAN (#AB1031 Merck Millipore, 1:100) and anti-RFP (#600-401-379, Rockland 1:250). FACS antibodies: anti-CD45 (#103131, BioLegend, 1:80), anti-Ter119 (#116227, BioLegend, 1:80), anti-CD31 (#562861, BD Pharmingen, 1:80), anti-CD200 (#ab33735, Abcam, 1:10), APC/Cy7 Streptavidin (#405208, BioLegend, 1:300).

Validation

All antibodies are validated, commercially available products that additionally have been validated in previously published studies (e.g. PMID: 21895533, PMID: 21264318, PMID: 33469143 & PMID: 25043045).

Animals and other research organisms

Policy information about studies involving animals; ARRIVE guidelines recommended for reporting animal research, and Sex and Gender in Research

Laboratory animals

Mice were maintained in the South Australia Health and Medical Research Institute (SAHMRI) Bioresources facility and Institute of Comparative Medicine at Columbia University, in accordance with JAX USA animal husbandry protocols. Animals were given food and water ad libitum and housed in temperature- (kept between 19°C - 24°C with an average temperature of 21°C - 22 °C), moisture-(humidity is kept between 45-55%), and light-controlled (12h light/dark cycle) individually ventilated cage systems.

LepR-cre (DeFalco et al., 2001, Science), Acan-creERT (Henry et al., 2009), Grem1-creERT (Worthley et al., 2015 Cell), R26-LSL-TdTomato (Madisen et al., 2010 Nat Neurosci), R26-LSL-ZsGreen (Madisen et al., 2010 Nat Neurosci) and R26-LSL-iDTR were imported from Jackson Laboratory and bred within the South Australia Health and Medical Research Institute (SAHMRI) Bioresources facility and Institute of Comparative Medicine at Columbia University. Grem1-TdTomato-iDTR mice (Grem1-creERT;DTR) were generated by mating homozygous Grem1-TdT mice to homozygous Rosa-iDTR. Acan-iDTR mice (Acan-creERT;DTR) were generated by mating heterozygous Acan-creERT mice to homozygous or heterozygous Rosa-iDTR. We observed leaky activity of the LepR-cre when the cre allele was maternally inherited and so the LepR-cre allele was maintained as a homozygote stock in males to breed to female mice WT for the locus to generate heterozygous LepR-cre/+ mice for lineage tracing experiments.

10 – 13 weeks old Grem1-TdT, Acan-TdT, LepR-TdT and C57BL/6 male mice were used to examine lineage tracing in health and DMM surgery model. Postnatal Day 4 – 6 (neonatal) and 6 weeks old (adult) Grem1-TdT, Acan-TdT and LepR-TdT mice were used to examine lineage tracing in early development and adulthood. 8 weeks old Grem1-TdT mice were given tamoxifen at 6 weeks of age to examine in vitro stem cell properties. 26 week old Grem1-creERT;FOXO1 mice were given tamoxifen at postnatal Day 4 – 6 (neonatal) and 6 weeks of age (adult) to examine the AC impact of FOXO1 deletion in Grem1 cells. 5 – 6 week old Grem1-TdT mice were given tamoxifen for ten days, then harvested at 7-9 weeks of age for scRNAseq analyses. 4 – 6 week old Grem1-TdT mice were used to examine the treatment of Fgf18 in AC with or without CiOA. 4 – 7 week old Grem1-Td-DTR, Acan-creERT;DTR and Grem1-creERT;DTR mice were used to examine the impact of the ablation of Grem1-lineage and Acan-lineage cells on AC.

Wild animals

This study did not involve wild animals.

Reporting on sex

Animal gender was not analysed as a variable during experiments for this study as, for this initial study into the role of chondrocytic precursor populations marked by Grem-1, we focused on phenotypes that were agnostic to sex. Future studies would benefit from larger cohort sizes to enable analysis of sex-specific phenomena. For this study, approximately equal numbers of male and female mice were used across experimental groups and for scRNAseq experiments.

Field-collected samples

This study did not involve samples collected from the field.

Ethics oversight

All animal experiments were conducted under approved protocols by the Animal Ethics Committees at the SAHMRI and Columbia University.

Note that full information on the approval of the study protocol must also be provided in the manuscript.

Flow Cytometry

Plots

Confirm that:

- [x] The axis labels state the marker and fluorochrome used (e.g. CD4-FITC).
- [x] The axis scales are clearly visible. Include numbers along axes only for bottom left plot of group (a 'group' is an analysis of identical markers).
- [x] All plots are contour plots with outliers or pseudocolor plots.
- [x] A numerical value for number of cells or percentage (with statistics) is provided.

Methodology

Sample preparation	Detailed sample preparation protocol is provided in methods section of the manuscript.
Instrument	Flow cytometry was performed on FACSFusion Cell sorter or LSR Fortessa (BD Biosciences).
Software	Flo Jo (v10.6.2) was used for data acquisition,
Cell population abundance	Quantification of cell populations is provided as a percentage of parent population as described in the gating strategy diagram.
Gating strategy	Gating strategy is provided in diagrams in manuscript.

- [x] Tick this box to confirm that a figure exemplifying the gating strategy is provided in the Supplementary Information.
